# Effect of electrospinning parameters on morphological properties of PVDF nanofibrous scaffolds

**DOI:** 10.1007/s40204-017-0071-0

**Published:** 2017-09-11

**Authors:** Asma Sadat Motamedi, Hamid Mirzadeh, Fereshteh Hajiesmaeilbaigi, Shadab Bagheri-Khoulenjani, MohammadAli Shokrgozar

**Affiliations:** 1 Biomedical Engineering Department, Amirkabir University of Technology (Tehran Polytechnic), Tehran, Iran; 2 Polymer and Color Engineering Department, Amirkabir University of Technology (Tehran Polytechnic), Tehran, Iran; 3 Optics and Quantum Technologies Research School, NSTRI, Tehran, Iran; 4 National Cell Bank of Iran, Pasteur Institute of Iran, Tehran, Iran

**Keywords:** Electrospinning, Morphology, PVDF nanofibrous scaffolds, Processing parameters, Tissue engineering

## Abstract

Smart materials like piezoelectric polymers represent a new class of promising scaffold in neural tissue engineering. In the current study, the fabrication processing parameters of polyvinylidine fluoride (PVDF) nanofibrous scaffold are found as a potential scaffold with nanoscale morphology and microscale alignment. Electrospinning technique with the ability to mimic the structure and function of an extracellular matrix is a preferable method to customize the scaffold features. PVDF nanofibrous scaffolds were successfully fabricated by the electrospinning technique. The influence of PVDF solution concentration and other processing parameters like applied voltage, tip-to-collector distance, feeding rate, collector speed and the solvent were studied. The optimal parameters were 30 w/v% PVDF concentration, 15 kV applied voltage, 18 cm tip-to-collector distance, 0.5 ml/h feeding rate, 2500 rpm collector speed and *N*,*N′*-dimethylacetamide/acetone as a solvent. The mean fiber diameter of the obtained scaffold was 352.9 ± 24 nm with uniform and aligned morphology. Finally, the cell viability and morphology of PC-12 cells on the optimum scaffold indicated the potential of PVDF nanofibrous scaffold for neural tissue engineering.

## Introduction

Nerve regeneration is a localized and complex biological phenomenon that prevents or restricts treatment in patients suffering from nervous system injuries. In this way, neural tissue engineering is a promising approach that has gained considerable interest (Gu et al. 2014). In the tissue engineering of central and peripheral nervous system, the characteristics of a scaffold as well as other tissues should be accounted for so as to emulate both function and morphology of the native tissue (Cao et al. 2009; Gu et al. 2014). Therefore, one of the main requirements to accomplish a tissue engineering process is to mimic cellular microenvironment to be appropriate for a specific targeted tissue (Pfister et al. 2011). In this regard, several studies have shown that porosity, topography, geometry, density, and mechanical properties of the scaffold besides its chemical and biological characteristics have dominant roles in cell enduring processes (Kim et al. 2012). During the last several decades, a variety of fabrication techniques including photolithography, phase separation, colloidal lithography, 3D printing, self-assembly and electrospinning have been studied to fabricate an ideal 3D scaffold with nano/micro features (Schmidt and Leach 2003). Among these techniques, electrospinning of the polymers into fibrous meshes with combination of nanoscale and microscale fibers has exhibited close physical characteristics of the extracellular matrix of cells. Electrospinning has been widely studied in neural tissue engineering due to its flexible setup which can be modulated depending on the polymer’s and scaffold’s features (Greiner and Wendorff 2007; Lee and Arinzeh 2011; Pham et al. 2006; Saeed et al. 2015).

A wide spectrum of synthetic and natural polymers has been studied in electrospinning of scaffolds in neural tissue engineering (Ai et al. 2013). Among synthetic polymers, biodegradable polymers like polyglycolic acid (PGA), poly(*l*-lactic acid) (PLLA), poly(lactic-*co*-glycolic) (PLGA), polyethylene glycol (PEG) and poly(ε-caprolactone) (PCL) are the most commonly studied (Hu et al. 2016). The influence of randomness, alignment, fiber diameter and surface modification of electrospun fibers on neural cell behavior have been investigated in several studies. These studies has demonstrated that the electrospun scaffold holds considerable potential to combat nerve regeneration (Gupta et al. 2009). Despite promising results, research works on finding innovative strategies for smart and active scaffolds have been conducted by various groups. In particular, since electric charges influence neurite extension and neuronal function, using electrically conductive polymers instead of conventional polymers has gained recent interest. Polypyrrole (PPy) and polyaniline (PANI) are two of the most studied conductive polymers that can assist electrically to stimulate and control neuron activities (Zhou et al. 2016). Piezoelectric polymers are another class of electroactive polymers that can produce electrical signals without any need for external stimuli (Prabhakaran et al. 2011). Among all polymers, polyvinylidene fluoride (PVDF) and polyvinylidene fluoride-trifluoroethylene (PVDF-TrFE) with piezoelectric coefficient of 24–34 and 38 PC/N subsequently show the highest electroactive properties (Correia et al. 2014; Weber et al. 2010). These piezoelectric materials have the ability to generate electrical charges in response to direct piezoelectric effect and mechanical stimulation; reversibly, they can be deformed by an electric stimulation (reverse piezoelectric effect). In fact, piezoelectric materials can induce an electrical field across their boundaries under mechanical stress, or vice versa (Ramadan et al. 2014). In biomedical applications, piezoelectric materials allow for the delivery of an electrical stimulus without the need of an external power source. Thus, the incorporation of these properties in bioscaffolds could be a way to obtain active bioscaffolds (Lhoste and http://tel.archives-ouvertes.fr/tel-00790208/ 2012). PVDF is a semi-crystalline polymer which started to draw scientific interest in the 1970s, because of its extraordinary pyroelectric, ferroelectric and piezoelectric properties (Richardson 1989). These properties with a combination of high elasticity and good processability lend this material to numerous technological applications. Another feature that distinguishes PVDF from other polymers is its polymorphism, that is, it may present at least five crystalline phases, namely α, β, γ and ε (Abdelaziz 2004). Among them, the α-phase is non-polar, whereas the other phases are electroactive (Lhoste and http://tel.archives-ouvertes.fr/tel-00790208/ 2012; Mandal et al. 2012). In addition, as a result of its good biocompatibility, PVDF also finds potential applications in the biomedical area (Damaraju et al. 2013; Ahmed et a1. 2013). Studies on PVDF and its copolymers for tissue engineering applications are mostly focused on bone, muscle and neural regeneration (Rajabi et al. 2015). Also, there are some studies on electrospinning of PVDF and its copolymers for biomedical and other applications (Baqeri et al. 2015; Cozza et al. 2013; Song 2016; Wang et al. 2014). Concerning nerve regeneration, the increase in the content of β-phase crystals of the PVDF scaffold would enhance its piezoelectricity while supporting neural cell growth (Lee et al. 2011). Since the complexity of nerve regeneration studies requires more reliable information on the relationship between scaffold design and neural cell behavior, in this study the electrospinning parameters such as solvent, applied voltage, injection rate, tip-to-collector distance and collector rotational speed are investigated in detail with respect to the PVDF scaffold architecture. The biocompatibility and morphology of neural cells in the optimized scaffold was also evaluated.

## Materials and methods

### Materials

Polyvinylidene fluoride (PVDF) pellets (MW = 84,000 Da) were procured from Sigma-Aldrich, and high purity acetone, *N*,*N′*-dimethylacetamide (DMAC) and *N*,*N′*-dimethylformamide (DMF) were purchased from Merck. Prior to the electrospinning process, different polymeric solutions at 10, 20, 25 and 30 (w/v%) PVDF concentrations were prepared by dissolving PVDF pellets in DMAC/acetone mixture of solvents in equal ratio to obtain neat polymeric PVDF solutions. Then, the mixtures were stirred further under constant and continuous magnetic stirring for approximately 2 h at 70 °C separately, without adding any catalyst, to allow PVDF dissolve completely and the optimum viscosity was obtained for the electrospinning process.

### Electrospinning process

Each resulting solution was transferred into a 5 ml glass syringe and loaded into the electrospinning apparatus (3 syringes, Full Option Lab 2 ESI-II, Nano Azma Company, Tehran, Iran). Electrospinning of the solutions was performed at an ambient temperature, with an applied voltage of 10–20 kV DC, injection rate of 0.3–0.7 ml/h, tip-to-collector distance (TCD) of 140–200 mm and mandrel rotational speed of 1000–3000 rpm for 15 min throughout the fiber collection process. The nanofibers were collected on the commercial aluminum foils and maintained at an ambient condition for further characterization.

### Characterization

#### SEM

The morphology and microstructure of the electrospun nanofiber mats were analyzed using scanning electron microscopy (SEM; AIS2100, Seron Technology, Korea) which accelerated at a voltage of 0.5–30 kV. The samples were previously sputter-coated with a thin layer of gold. To measure the diameters of the nanofibers and the magnitude of their distribution, the Digimizer software (version 4.6.1) was used, obtaining 50 individual nanofiber microscopy images, twice under the same conditions.

#### XRD

To evaluate the crystalline phase of the PVDF nanofibrous mat, it was analyzed by wide angle X-ray diffractometer (XRD; Panalytical, X’ PertPro) using a Cu radiation source with a wavelength of 1.54 Å working at 40 kV and 40 mA. The intensity was recorded in the range of 5°–80° by a step size of 0.026°.

#### FTIR

To characterize the crystalline phase and piezoelectricity within the nanofibers, Fourier transform infrared (FTIR) spectra of the PVDF nanofibrous mat was registered by a BOMEM (Canada) spectrometer with the functionality in the absorption mode. For this purpose, first the rubbed nanofibers were mixed with KBr powder and allowed to dry at 100 °C for 24 h before use. The weight ratio was 1/100 and the mixture was pressed to form a clear disc which was placed on the holder in front of the beam. FTIR spectra of the samples were measured in the range of 4000–400/cm with a resolution of 2/cm within 20 scans.

### Cell culture

PC-12 cell lines derived from the rat adrenal medulla (NCBI, National Cell Bank of Iran) were cultured in high-glucose DMEM (Gibco, USA) supplemented with 10% (v/v) fetal bovine serum (FBS, Gibco, USA) and 1% penicillin/streptomycin (100 U/ml penicillin and 100 µg/ml streptomycin) and incubated at 37 °C in a humid atmosphere with 5% CO_2_.

For cell adhesion and cell morphology study, the prepared nanofibrous mats (cut into 2 × 3 cm^2^) were sterilized by gamma radiation (within a dose of 25 kGy/h) and the PC-12 cells were seeded onto the scaffolds with a density of 1 × 104. After being incubated for 24 h, adherent cells were fixed using 2.5% glutaraldehyde and dehydrated serially by immersion in 30–100% ethanol solutions. The morphology of the fixed cells cultured on the scaffolds was examined by SEM images.

For cell viability study, the prepared sterilized nanofibers were exposed to 3 ml serum-free culture medium at 37 °C for 7 and 14 days to obtain the extracts of the samples. PC-12 cells were seeded in 96-house culture plates with the density of 1 × 10^4^ cells/well, and 100 μl of RPMI culture medium containing 10% FBS (fetal bovine serum) was added to each cell-house. After incubating for 24 h, the medium was replaced by sample extracts. Once again, the cultured plates were incubated for 24 h. The culture medium without the extracts of the samples was considered as the control. For quantification of the cells’ viability, tetrazolium dye salt, 3-[4,5-dimethylthiazol-2-yl]-2,5-diphenyltetrazolium bromide (MTT Sigma, USA) assay, was carried out. MTT solution (0.5 mg MTT powder/ml FBS) of 100 ml was added to each cell-house. After incubating for 4 h at 37 °C, 100 µl DMSO was added into each well and placed in a shaker incubator at 37 °C for 15 min until all formazan crystals were dissolved. Next, 100 µl of the solution from each well was transferred into another 96-well tissue culture plate and light absorbance was measured at 545 nm with a multiwall microplate reader (STAT FAX 2100, USA). The MTT test was repeated three times for each sample and normalized with tissue culture polystyrene (TPS) as the negative control and the mean value is reported as the mean ± STVD.

## Results and discussion

### Morphological studies, fiber size and distribution

**Fig. 1:**
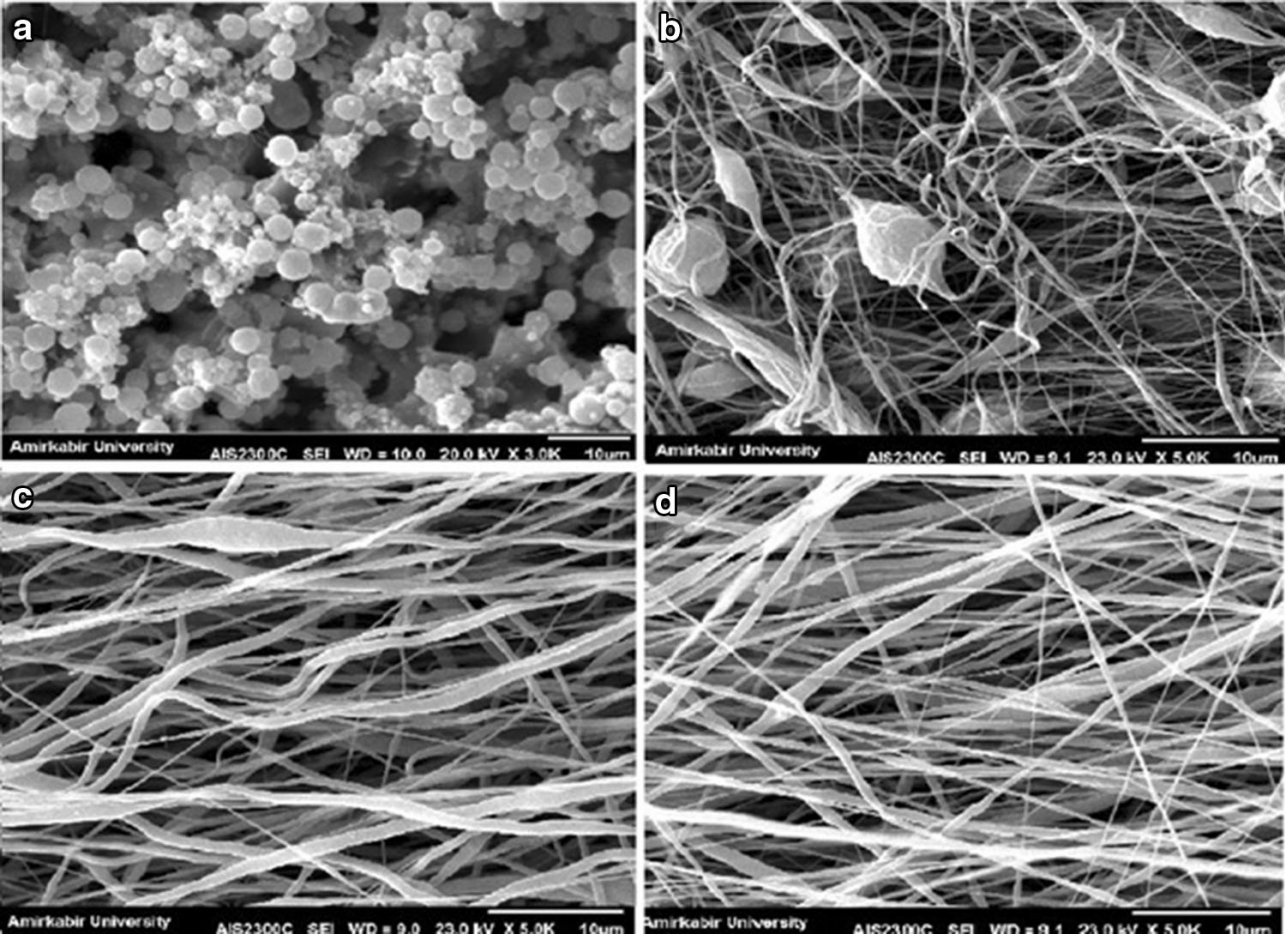
SEM images of PVDF nanofibers with different solution concentrations: **a** 10%, **b** 20%, **c** 25% and **d** 30%

In this study, six independent variables including PVDF concentration, applied voltage, tip-to-collector distance, feeding rate, collector speed and the solvent were selected and their effects on mat morphology and mean fiber diameters were investigated. To reach a significant conclusion, each variable was compared to other variables in a separate table and the SEM images were also categorized in separate pictures. According to Table 1, the first variable is PVDF concentration at 10, 20, 25 and 30 (w/v%), while other parameters were constant. In fact, to achieve fine and decent fibers through electrospinning, an adequate amount of polymer with suitable molar weight is crucial. As it is clear in SEM images in Fig. 1, at low concentration, electrospraying happens and PVDF microparticles are formed. When using the solution of 20% concentration, the structure is composed of non-uniform electrospun fibers together with some large beads in between. Increasing the PVDF concentration to 25 wt% has led to the formation of bead-less electrospun structure. But the diameter and orientation of the observed nanofibers are not completely uniform. At 30% concentration, it seems that more uniform fibers have been formed. This may be due to the increasing amount of polymer in electrospinning jet and more interactions between polymer chains in solution, which later results in greater resistance of the solution against the pulling force by electrical charges (Sukigara et al. 2003). In addition, with the increase of PVDF concentration, the mean fiber diameters also increased. Accordingly, this concentration (30%) was considered for further structural optimization of PVDF electrospinning.

**Table 1:** Mean diameters of PVDF nanofibers with different solution concentrations measured from SEM micrographs

Sample	Solvent	Concentration (w/v%)	Voltage (kV)	Distance (cm)	Feeding rate (ml/h)	Collector speed (rpm)	Mean nanofiber diameter (nm)
S1	DMAC/acetone	10	15	14	0.7	1000	***
S2	DMAC/acetone	20	15	14	0.7	1000	***
S3	DMAC/acetone	25	15	14	0.7	1000	472.9 ± 21
S4	DMAC/acetone	30	15	14	0.7	1000	644.2 ± 29

**Fig. 2:**
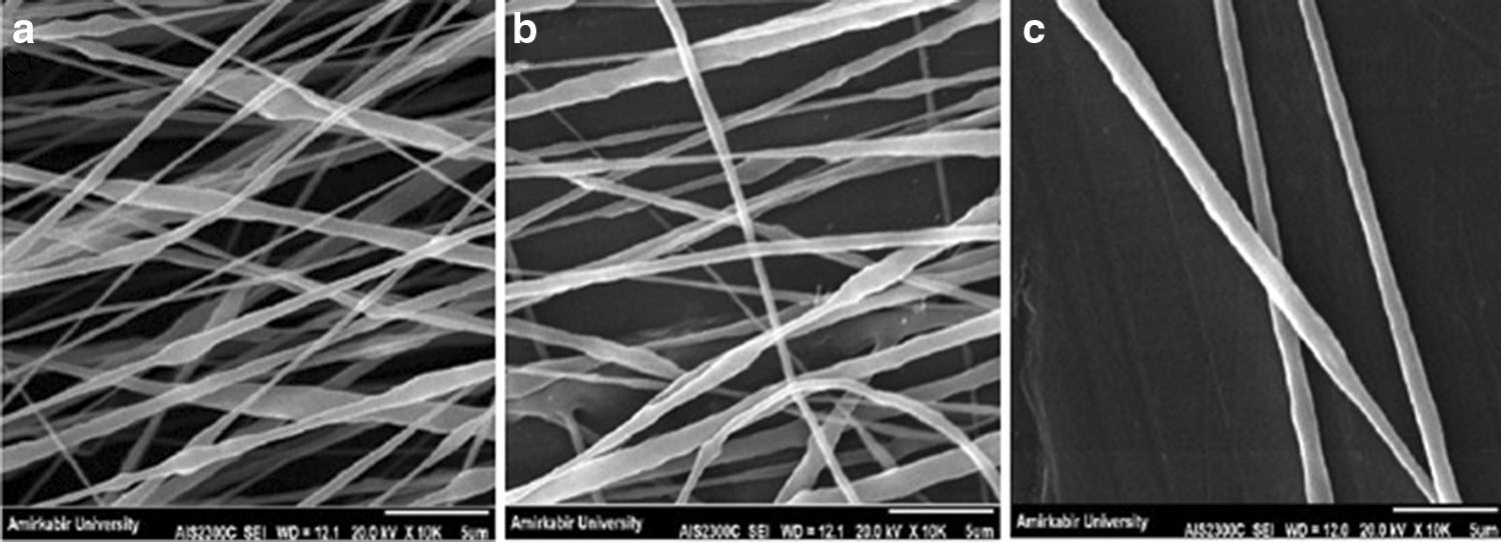
SEM images of PVDF nanofibers with different voltages: **a** 10 kV, **b** 15 kV and **c** 20 kV

The fundamental principle of electrospinning is based on a high voltage injected into the polymer solution. In this process, the electrostatic force overcomes the surface tension of the polymer causing the initiation of e jet (Lee and Arinzeh 2011). According to Table 2 and Fig. 2, the effect of varying the applied voltage (10, 15 and 20 kV) on the fiber morphology and diameter was studied. Significant differences were detected in fiber morphology and mean fiber diameter for 10 and 20 kV, respectively. The fibers have smaller diameters at lower voltage and bead formation occurs because the applied voltage has a direct effect on fiber elongation and solvent evaporation. On the other hand, at higher voltage, the polymer jet becomes highly unstable and produces thicker fibers with a large variation in size that can be seen in high standard deviation of the fiber diameter.

**Table 2:** Mean diameters of PVDF nanofibers with different voltages measured from SEM micrographs

Sample	Solvent	Concentration (w/v%)	Voltage (kV)	Distance (cm)	Feeding rate (ml/h)	Collector speed (rpm)	Mean nanofiber diameter (nm)
S5	DMAC/acetone	30	10	14	0.7	1000	308.4 ± 11
S4	DMAC/acetone	30	15	14	0.7	1000	644.2 ± 29
S6	DMAC/acetone	30	20	14	0.7	1000	855.1 ± 34

**Fig. 3:**
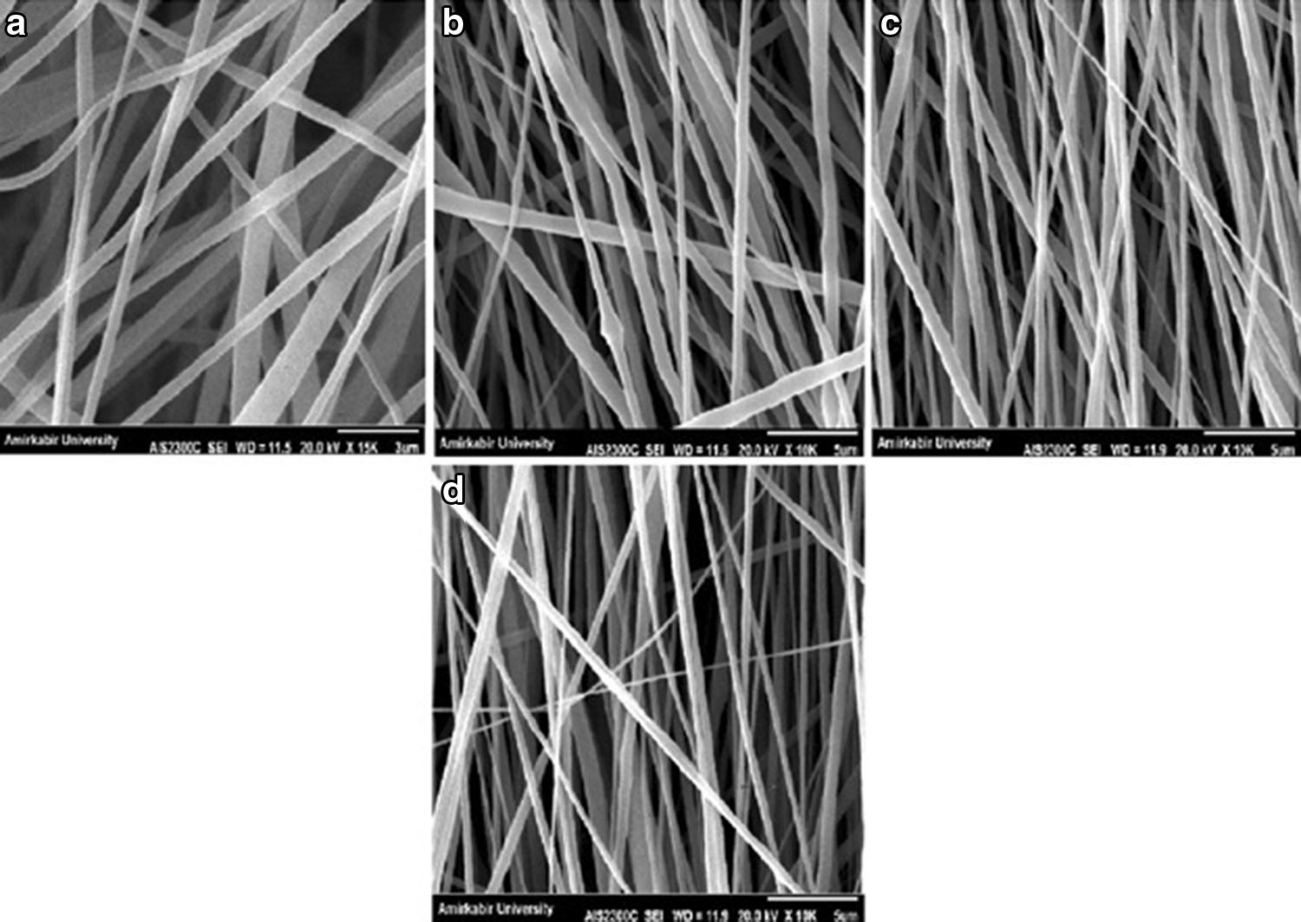
SEM images of PVDF nanofibers with different tip to collector distances: **a** 18cm, **b** 20 cm, **c** 22 cm and **d** 24 cm

Tip-to-collector distance is another parameter which has influence on the mean fiber diameter and morphology. In fact, the bead formation prevention is attributed to the complete solvent evaporation at optimal distance. The selected tip-to-collector distances were 14, 16, 18 and 20 cm. As observed in SEM images of Fig. 3, there is no significant difference in fiber morphology for various distances. Similar results have been reported in the spinning of gelatin, chitosan, poly(vinyl alcohol) and poly(vinylidene fluoride) (Pham et al. 2006). Nevertheless, the mean fiber diameter shows a descending trend and with increasing the distance the mean fiber diameter decreases (Table 3).

**Table 3:** Mean diameters of PVDF nanofibers with different tip-to-collector distances measured from SEM micrographs

Sample	Solvent	Concentration (w/v%)	Voltage (kV)	Distance (cm)	Feeding rate (ml/h)	Collector speed (rpm)	Mean nanofiber diameter (nm)
S4	DMAC/acetone	30	15	14	0.7	1000	644.2 ± 29
S7	DMAC/acetone	30	15	16	0.7	1000	509.8 ± 24
S8	DMAC/acetone	30	15	18	0.7	1000	432.2 ± 21
S9	DMAC/acetone	30	15	20	0.7	1000	264.4 ± 16

**Fig. 4:**
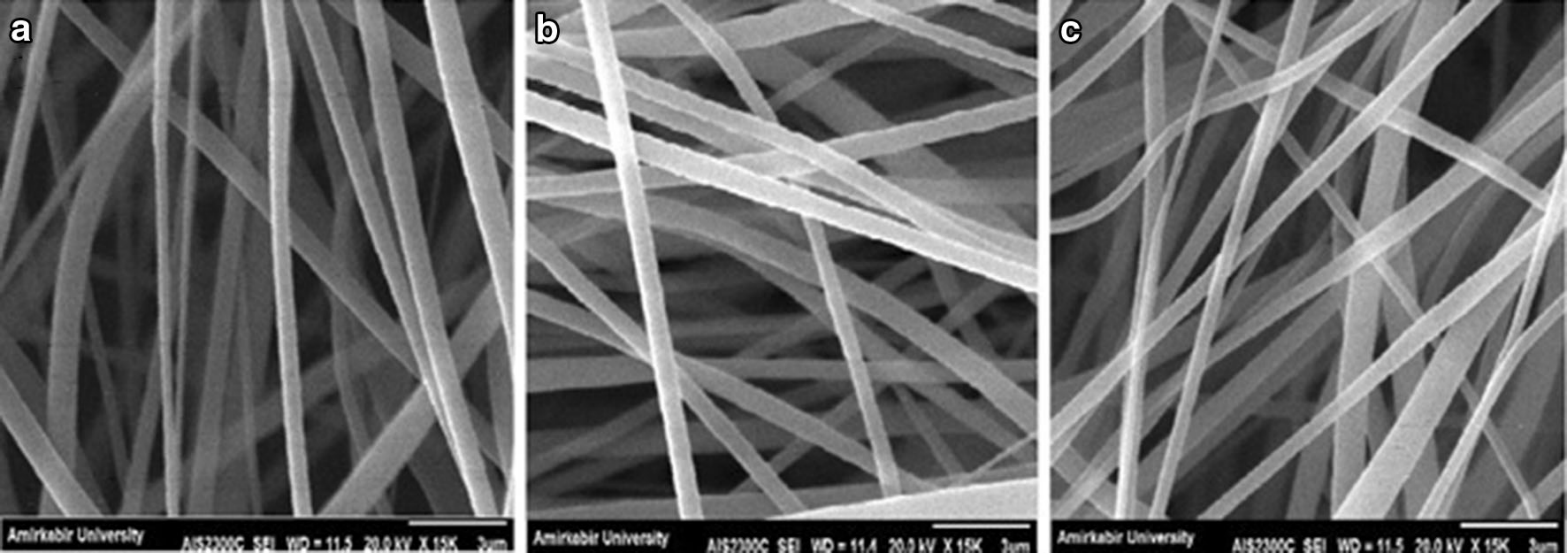
SEM images of PVDF nanofibers with different feeding rates: **a** 0.3 ml/h, **b** 0.5 ml/h and **c** 0.7 ml/h

It is clear that the feeding rate determines the amount of polymer solution available for electrospinning. As a result, lower feeding rate produces fibers with smaller diameter (Zong et al. 2002). The influence of feeding rate on the mean fiber diameter and morphology is shown in Table 4 and Fig. 4. In this study, 0.3, 0.5 and 0.7 ml/h feeding rates are selected for the above evaluation. As expected, by increasing the feeding rate the mean fiber diameters increase. According to the SEM images in Fig. 4, the feeding rates are not too high for bead formation to take place. Under high feeding rates, the fibers do not dry completely prior to reaching the collector.

**Table 4:** Mean diameters of PVDF nanofibers with different feeding rates measured from SEM micrographs

Sample	Solvent	Concentration (w/v%)	Voltage (kV)	Distance (cm)	Feeding rate (ml/h)	Collector speed (rpm)	Mean nanofiber diameter (nm)
S10	DMAC/acetone	30	15	18	0.3	1000	365.6 ± 13
S11	DMAC/acetone	30	15	18	0.5	1000	520.6 ± 14
S8	DMAC/acetone	30	15	18	0.7	1000	432.2 ± 21

**Fig. 5:**
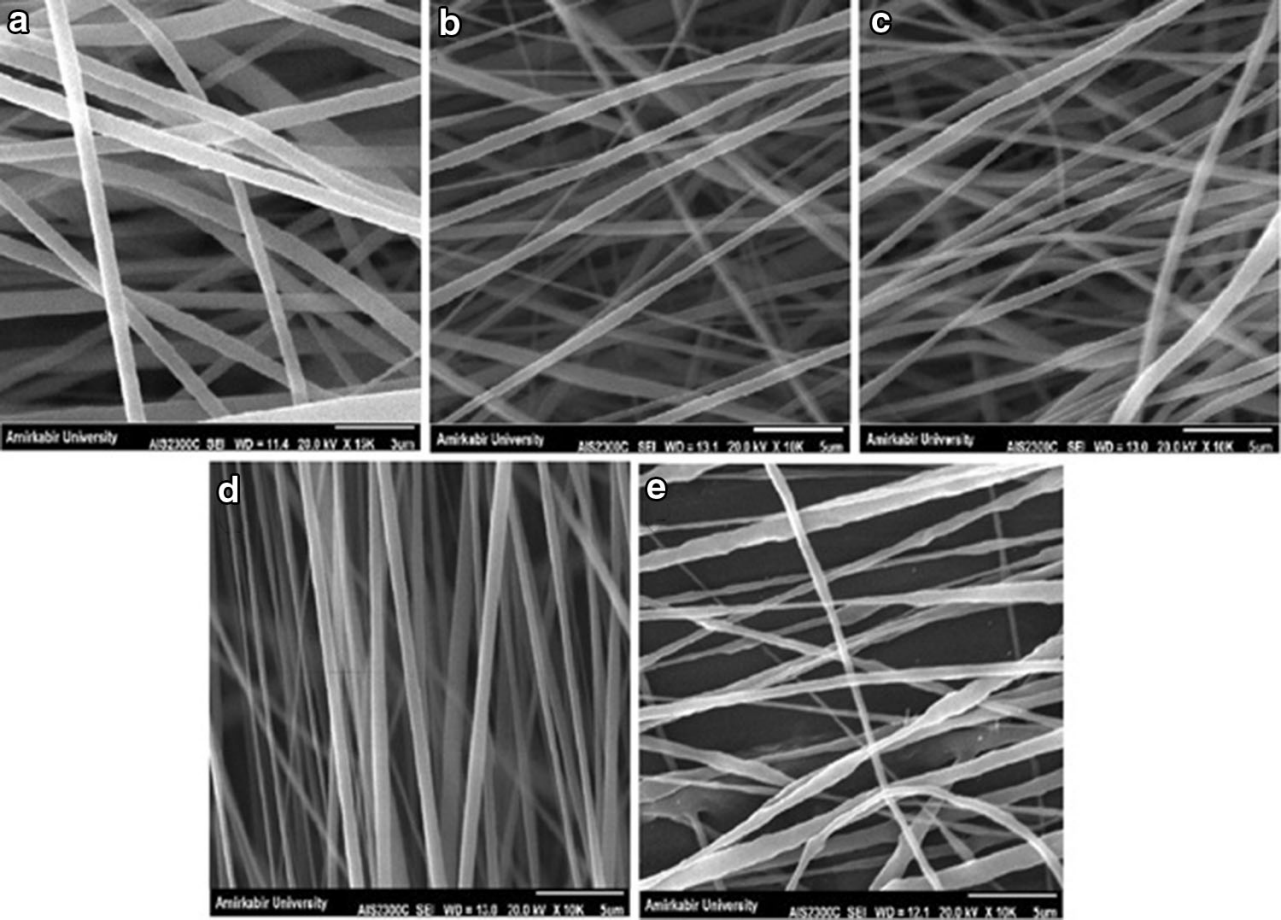
SEM images of PVDF nanofibers with different collector speeds: **a** 1000 rpm, **b** 1500 rpm, **c** 2000 rpm, **d** 2500 rpm and **e** 3000 rpm

Contact guidance is a well-known phenomenon in nerve regeneration and in several studies has been considered as an important clue for peripheral nerve regeneration (Chew et al. 2008; Gupta et al. 2009). In fact, the aligned nanofibers lead to neurite outgrowth parallel to the fiber directions, which results in preferable nerve repair. In this study, to provide aligned nanofibrous scaffold, a high-speed rotating disk was set with 1000, 1500, 2000, 2500 and 3000 rpm rotation speeds. The rotating disk allows the fiber to collect along the direction of rotation, because the rotating disc exerts a tangential force on the polymer jet to stretch and align the resulting fibers. By increasing the collector speed to 2500 rpm, the nanofibers show a descending trend in the mean fiber diameter. Moreover, as shown in the SEM images in Fig. 5, the fibers are well aligned at 2500 rpm. But at higher speed, the mean fiber diameters increase and the scaffold has non-uniform fibers with less alignment because of fiber discontinuity (Fig. 5; Table 5).

**Fig. 6:**
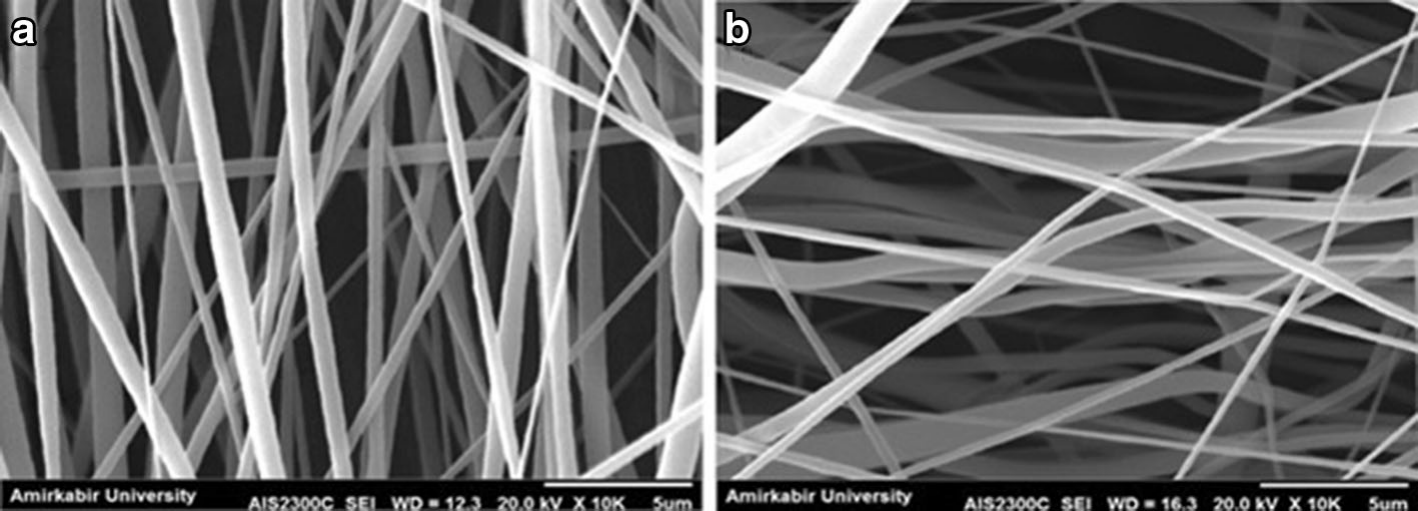
SEM images of PVDF nanofibers with different solvent systems: **a** DMAC/Acetone and **b** DMF/Acetone

**Table 5:** Mean diameters of PVDF nanofibers with different collector speeds measured from micrographs

Sample	Solvent	Concentration (w/v%)	Voltage (kV)	Distance (cm)	Feeding rate (ml/h)	Collector speed (rpm)	Mean nanofiber diameter (nm)
S11	DMAC/acetone	30	15	18	0.5	1000	520.6 ± 14
S12	DMAC/acetone	30	15	18	0.5	1500	511.8 ± 24
S13	DMAC/acetone	30	15	18	0.5	2000	313.0 ± 20
S14	DMAC/acetone	30	15	18	0.5	2500	352.9 ± 24
S15	DMAC/acetone	30	15	18	0.5	3000	462.5 ± 19

To verify the selected electrospinning evaluation results, the optimal parameters were chosen and compared with the new solvent. Therefore, new samples were electrospinned at 30 w/v% PVDF concentration, 15 kV applied voltage, 18 cm tip-to-collector distance, 0.5 ml/h feeding rate and 2500 rpm collector speed. Generally, PVDF is soluble in some common solvents like *N*-methyl-2-pyrrolidone (NMP) (boiling point 202 °C), DMF (152 °C), DMAC (boiling point 165 °C) and acetone (boiling point 56 °C). The slower evaporation rate of solvent may allow fibers to undergo high elongation which introduces high strain rates. These strains can extend and align polymer chains and promote the transformation of α phase to β phase. On the other hand, inefficient solvent evaporation results in bead formation. In this regard, according to SEM images, acetone improved the solvent evaporation rate during the electrospinning process. Besides the evaporation rate, the solvent polarity plays a main role in the crystal structure of the polymer. It is well known that for semi-crystalline polymer, different solvents with different polarities will induce different crystalline structures. As a result, solvents of different nature will change the piezoelectricity of PVDF mats. Tian et al. (2006) have shown that DMAc produces γ phase because of high solvent polarity. According to Table 6, the solvents are compared together, while other variables are kept constant. By replacing the DMAC with DMF, the nanofiber diameters are increased unfavorably. The SEM images of PVDF nanofibers with different solvent systems are shown in Fig. 6.

**Table 6:** Mean diameters of PVDF nanofibers with different solvent systems measured from SEM micrographs

Sample	Solvent	Concentration (w/v%)	Voltage (kV)	Distance (cm)	Feeding rate (ml/h)	Collector speed (rpm)	Mean nanofiber diameter (nm)
S14	DMAC/acetone	30	15	18	0.5	2500	352.9 ± 24
S16	DMF/acetone	30	15	18	0.5	2500	611.9 ± 27

### Crystalline structure studies

**Fig. 7:**
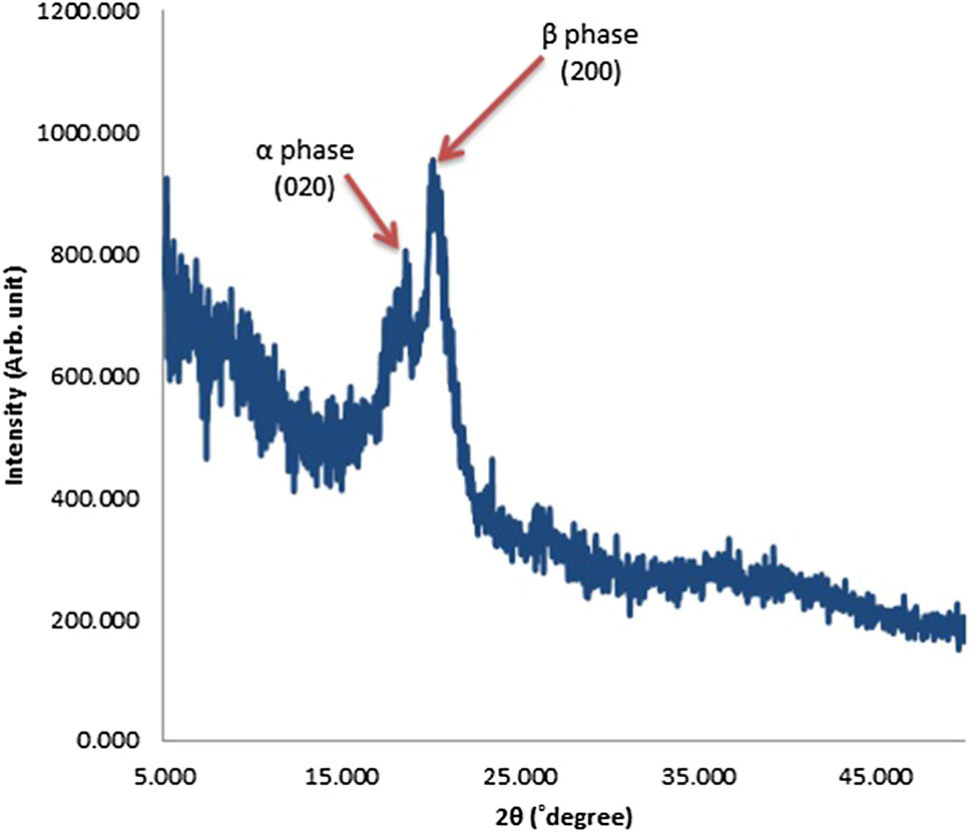
XRD pattern of PVDF nanofibers

The crystalline structure of PVDF polymers indicate α-, β- and γ-phases and each phase has specific diffraction peaks in the 2*θ* Bragg angle during XRD measurement (Motamedi et al. 2017). The XRD pattern of the optimized PVDF nanofibers (sample S14) is shown in Fig. 7, where the peaks at 18° (0 2 0) and 20° (2 0 0) correspond to α-phase and stand for the β-phase crystalline structure, respectively, as indicated in Fig. 7 (Mahato et al. 2015).

**Fig. 8:**
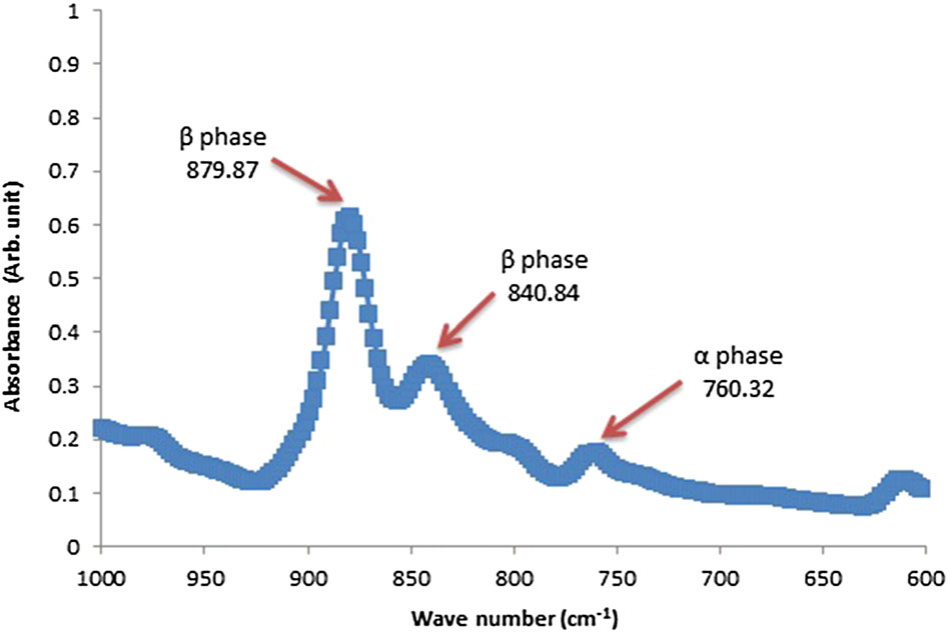
FTIR spectrum of PVDF nanofibers

Different crystalline phases absorb different infrared wavelengths; thus, FTIR can be used to identify crystalline phases (Motamedi et al. 2017). The FTIR spectrum of PVDF nanofibers mat is shown in Fig. 8. The most significant peaks corresponding to α- and β-phases are indicated on the spectrum (Mahato et al. 2015). It is known that the piezoelectric properties come from the β-phase of the PVDF(Devikala et al. 2013).

As demonstrated in Fig. 8, the characteristic peak of β-phase which refers to the piezoelectric crystalline phases of PVDF nanofibers, appears at 880/cm.

### Cell viability and morphological studies

**Fig. 9:**
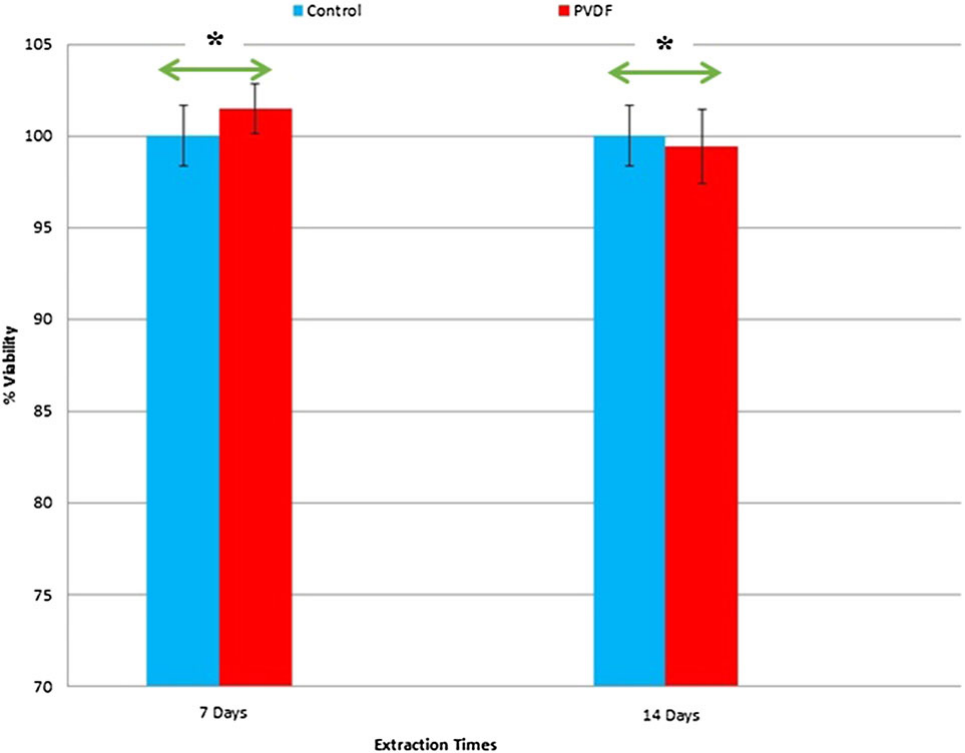
The viability of PC-12 cells in PVDF nanofibrous mats extractions on days 7 and 14 comparison to control group (*; P value less than 0.05)

**Fig. 10:**
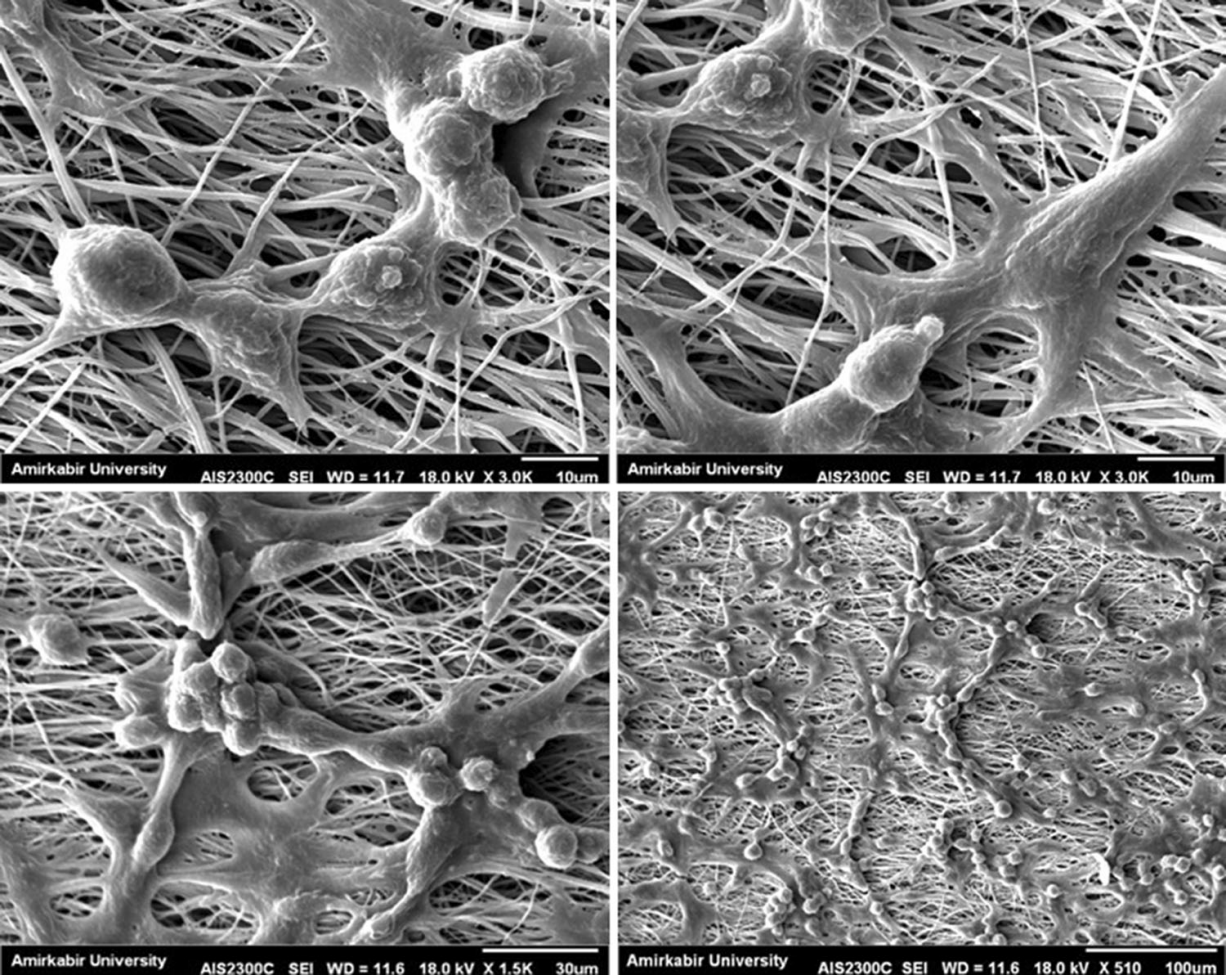
SEM micrographs of PC-12 cells seeded and attached on PVDF nanofibers after 1 day with different magnifications

The viability of PC-12 cells seeded on culture plate with and without PVDF nanofibrous mats extractions on days 7 and 14 was evaluated by an MTT assay (Fig. 9). For this purpose, PVDF nanofibers obtained under optimized electrospinning conditions of a voltage of 15 keV, distance 15 cm, feeding rate 0.5 ml/h and collector speed of 2500 rpm were more aligned, uniform and defectless nanofibers than the others (sample S14). PVDF nanofibrous mat extractions on day 7 in comparison with the control group show higher number of cells, while extraction on day 14 shows lower number of cells; however, there were no statistically significant differences (*p* < 0.05) among extractions and control groups. Thus, this result shows that the nanofibrous mat can provide a suitable environment for cell growth and does not affect cell viability and their proliferation rates. SEM images shown in Fig. 10 are recorded after 24 h of cell culturing. The cells are attached and well spread on the entire scaffold. It is clear that cells change from their original circular shape to outgrowth and elongated form. In images of higher magnification (Fig. 10), the direction of cell elongation and neurite spread is parallel to the direction of fibers which is valuable for cell–scaffold interactions in nerve regeneration strategies (Lins et al. 2016).

## Conclusion

Tuned scaffolds have displayed an effective approach toward neural tissue engineering. Electrospinning shows a great potential for fabrication of designed scaffolds. On the other hand, combination of electrospinning with new smart materials, like piezoelectric polymers, opens a new era in neural tissue engineering. In this way, PVDF nanofibrous mats were prepared by electrospinning, and to obtain a favorable scaffold, studies were focused on electrospinning parameters. It is found that several parameters have influence on the mean fiber diameter and morphology including the solvent, PVDF concentration, applied voltage, tip-to-collector distance, feeding rate and collector speed. In particular, the PVDF solution concentration, applied voltage and collector speed have been shown to influence the fiber size and morphology significantly. With optimization of these parameters, a favorable scaffold with nanoscale morphology and microscale alignment was achieved. On the other hand, based on the cell viability and behavior studies, this scaffold shows potential as a neural tissue engineering scaffold. Future studies will focus on the effect of processing parameters on piezoelectricity of the scaffold and their influence on cell behavior.
